# Atypical PKCs activate Vimentin to facilitate prostate cancer cell motility and invasion

**DOI:** 10.1080/19336918.2021.1882782

**Published:** 2021-02-11

**Authors:** Wishrawana S. Ratnayake, Christopher A. Apostolatos, Sloan Breedy, Clare L. Dennison, Robert Hill, Mildred Acevedo-Duncan

**Affiliations:** a Department of Chemistry, University of South Florida, Tampa, FL, USA; b Department of Integrative Biology, University of South Florida, Tampa, FL, USA; c Department of Cell Biology, Microbiology and Molecular Biology, University of South Florida, Tampa, FL, USA

**Keywords:** PKC-ι, PKC-ζ, Vimentin dynamics, phosphorylation, metastasis, prostate cancer

## Abstract

Atypical protein kinase C (aPKC) are involved in progression of many human cancers. Vimentin is expressed during epithelial to mesenchymal transition (EMT). Molecular dynamics of Vimentin intermediate filaments (VIFs) play a key role in metastasis. This article is an effort to provide thorough understanding of the relationship between Vimentin and aPKCs . We demonstrate that diminution of aPKCs lead to attenuate prostate cellular metastasis through the downregulation of Vimentin expression. *si*RNA knocked-down SNAIL1 and PRRX1 reduce aPKC activity along with Vimentin. Results suggest that aPKCs target multiple activation sites (Ser33/39/56) on Vimentin and therefore is essential for VIF dynamics regulation during the metastasis of prostate cancer cells. Understanding the aPKC related molecular mechanisms may provide a novel therapeutic path for prostate carcinoma.

## Introduction

aPKCs contain two isoforms PKC-iota (PKC-ι) and PKC-zeta (PKC- ζ) and they solidly satisfy the criteria of an oncogenic kinase [1,2]. Overexpression of PKC-ζ was observed in several cancers such as prostate, lymphoma, melanoma and bladder cancers [2–6]. Win, *et al*. described that PKC-ζ induced malignant and transformed nonmalignant prostate cell survival by phosphorylating IKKαβ thereby promoting NF-κB activation [7]. Filomenko, *et al*. described that PKC-ζ is activated and provides protection for cancer cells in the presence of a cytotoxic agent; Etoposide (VP-16) [8]. Bezombes, *et al*. showed that elevated levels of PKC-ζ caused a hindered apoptosis which increased the resistance to antileukemic drugs such as daunorubicin and 1-beta-D-arabinofuranosylcytosine in U937 human leukemia cells [9]. Collecting evidence confirms the oncogenic properties of PKC which heavily contributes to the transformed phenotype multiple cancers [10,11]. Higher levels of PKC-ι are detected in several tumors, such as pancreas, lung, colon, breast, prostate, ovarian and melanoma [11]. In several carcinomas, the PRKCI gene is documented to be amplified, even if PKC-ι overexpression does not necessarily associate with gene amplification [10–16].

Vimentin is a crucial structural protein of the Type III intermediate filament (IF) protein family. IFs constitute an extensive grid of interlinking proteins between the nuclear envelope and the plasma membrane which provides mechanical support and transfer information between the nucleus and cell surface. Vimentin expression is unique to the cell type and tissue which often express in fibroblasts, cells with mesenchymal origin, lymphocytes and majority of tumor cells [17,18]. Vimentin plays a crucial role in cell polarity maintenance and polarity change, microfilament system organization and cellular motility. In fact, enhanced expression of Vimentin is a hallmark of EMT that plays an essential role in achieving rear-to-front polarity for mesenchymal cells. In addition to EMT, vimentin is observed in the growth, immune response and wound healing mechanisms of cells [19–22]. Multiple phosphorylation events are required for Vimentin activation. Protein kinase A (PKA), PKC, cyclin-dependent kinase 1 (CDK1), Ca^2+^/calmodulin-dependent protein kinase II (CaM kinase II), RhoA kinase, RAC-alpha serine/threonine-protein kinase (AKT1) and RAF proto-oncogene serine/threonine-protein kinases (Raf-1-associated kinases) have been established as Vimentin phosphorylating kinases [23,24]. Ser6, Ser7, Ser8, Ser33, Ser39, Ser56, Ser71, Ser72, and Ser83 serve as specific phosphorylation sites on Vimentin head region [18,23–30]. While Vimentin can be activated by phosphorylation it can also be deactivated through the means of phosphorylation [25]. The enzyme Rho-Kinase is found to phosphorylate Vimentin leading to the inhibition of its filament formation *in-vitro*. This inhibition occurs via phosphorylation of the Ser71 site on Vimentin. *In-vivo* Rho-kinase has been observed to translocate to the cleave furrow during cytokinesis [25]. When this translocation occurs, Rho-kinase phosphorylates Vimentin on Ser71 and this activity may contribute to the formation of the contractile ring during cytokinesis [25].

Although a thorough understanding of the molecular dynamics of Vimentin organization in relation to cell migration has not been achieved, VIFs have been found to interact with microtubules which are involved in the polarity maintenance for directional migration of cells. Microtubules are the master organizers of the epithelial cell’s apical-basal polarity maintenance [31,32]. During EMT and in mesenchymal cells, VIFs accumulate an ultra-structural grid on microtubules which are already polarized and control the growth and contraction of microtubules to a precise template by maintaining rear-front polarity which heightens the movement efficacy of cells. This is accomplished as VIF assembles alongside the microtubules to form a replica of the formerly polarized microtubule grid which has a slower rate of turn over. This is important as the orientation of microtubules is responsible for conferring the front-rear asymmetry which is characteristic of mesenchymal cells [31,32]. Gan, Z. *et al*. show that disruption of VIF network on microtubules leads to significant changes in the microtubule network and changes the polarity of cells [31]. Ivaska, J. *et al*. discussed the involvement of PKC-ε on phosphorylation of Vimentin and control of integrin recycling to facilitate cell motility [24]. Integrins are heterodimeric receptors of the cellular surface that facilitate adhesion and migration between cells and cell matrix. There are three major steps in motility; (I) construction of projection and new adhesion sites at top end (II) shrinkage of cell (III) detachment from trailing end. Phosphorylation of Vimentin is central in regulation of these dynamics and Vimentin expression correlates with increased migration following EMT [24]. Vimentin polymerization begins with two Vimentin protein chains interacting parallel to each other forming a homodimer. Eight homodimers then interact together to form unit length filaments (ULF) of Vimentin. These ULFs serve as the building blocks of VIFs and interact with each other in tandem to form longer Vimentin chains and later they undergo super compaction to form mature VIFs [33,34].

We established PKC-ι and PKC-ζ as oncogenes and identified as a prospective therapeutic target for melanoma and prostate carcinoma based on the aPKC specific inhibitors applications in recent times [19,20,35–38]. Results demonstrated that elevated levels of PKC-ι and PKC-ζ in prostate cancer cell lines (DU-145 and PC-3) heavily contributed to their progression at different levels [36]. Results summarized that both PKC-ι and PKC-ζ prompt cell survival via the NF-κB/PI3K/AKT pathways. Inclusion of TNF-α augmented the levels of aPKCs and thus increased the transfer of the activated NF-κB complex to the nucleus [36]. In the current study, we account the properties of aPKC attenuation on VIF dynamics in detail while giving emphasis to the regulation of expression of Vimentin. The changes in gene expression during EMT involve principal regulators such as Zinc finger protein SNAI1 (SNAIL1), Zinc finger E-box-binding homeobox1 (ZEB1), Twist-related protein1 (TWIST) and Paired related homeobox 1 (PRRX1). Here we knockdown the expression of these TFs using *si*RNA. These TFs have highly specific expression profiles and known to upregulate Vimentin expression during EMT [39–44]. Only PRRX1 and SNAIL1 demonstrated a connection to both aPKC and Vimentin expression profiles. We have also tested the degree of phosphorylation at Ser6, Ser33, Ser39, Ser56 and Ser71 on Vimentin upon aPKC attenuation. Data suggested that aPKC attenuation significantly downregulate Smad signaling. Our overall results recommended that both aPKCs are critical components in Vimentin dynamics in which they are involved in the phosphorylation of Vimentin at Ser33, Ser39 and Ser56 to significantly activate Vimentin, thereby leading to the disassembly of VIF to support prostate cancer cell metastasis. Results also indicated that aPKC attenuation retards Vimentin expression through downregulation of SNAIL1 and PRRX1.

## Materials and methods

### Materials

[4-(5-amino-4-carbamoylimidazol-1-yl)-2,3-dihydroxycyclopentyl] methyl dihydrogen phosphate (ICA-1 T) (Therachem, Jaipur, India) and 8-hydroxy-1,3,6-naphthalenetrisulfonic acid (ζ-Stat) (NSC 37044) was provided by National Institute of Health (NIH, Bethesda, MD, USA). Sterile distilled water was used as the solvent. Materials were acquired as follows; primary antibodies of PKC-ζ (sc-17781, Santa Cruz Biotech), PKC-ι (610175, BD Biosciences), p-PKC-ι (T555, 44–968 G), p-PKC-ζ (T410, PA5-17837) and E-Cadherin (701134, Thermo Fisher Scientific), Vimentin (5741S), p-Vimentin (S39, 13614S), p-Vimentin (S56, 7391S), p-Smad2 (S465/467)/Smad3 (S423/425) (8828S) and SNAIL1 (3879S, Cell Signaling Biotechnology). PRRX1 (ab211292) and p-Vimentin (S71, ab115189, Abcam). p-Vimentin (S6, ADI-KAM-CC245-E) and p-Vimentin (S33, ADI-KAM-CC246-E, Enzo Life Sciences). β-actin-peroxidase (A3854, Sigma). Enhanced chemiluminescence solution (34,080, Pierce Inc.). *si*RNA (human small interfering RNA) for PKC-ζ (SR321432), PKC-ι (SR321426), SNAIL1 (SR304489, OriGene Inc.) and PRRX1 (AM16708, Thermo Fisher Scientific). DPBS without Mg^2+^ and Ca^2+^ ions (Dulbecco’s phosphate-buffered saline, D8537) and Trypsin–EDTA (Ethylenediaminetetraacetic acid, T4049, Sigma Aldrich).

### Cell culture

DU-145 (ATCC® HTB-81™), PC-3 (ATCC® CRL-1435™), and RWPE-1 (ATCC® CRL-11609™) cells were obtained from ATCC (American Type Tissue Culture Collection; Rockville, MD, USA). All cell lines were authenticated by ATCC using karyotyping, morphology and PCR-based approaches. Early passages of cells were cryo-preserved in liquid nitrogen and cells of early passages were resuscitated from liquid nitrogen for experiments. 37°C and 5% CO_2_ were maintained as cell culture conditions. EMEM (ATCC 30–2003) and RPMI-1640 Media (ATCC 30–2001) were used with fetal bovine serum (FBS, 10% v/v) and Penicillin (5 µg/ml) for DU-145 and PC-3, respectively. K-SFM medium (17005–042) with FBS (10% v/v) and Penicillin (5 µg/ml) were used for RWPE-1 cells.

### Cell migration and invasion assays

The detailed procedure for wound healing assay was performed for prostate cancer cells as described in Ratnayake, *et al*. [20]. *si*RNA of PKC-ι or PKC-ζ (20 nM) treatments were conducted against scrambled *si*RNA for 3 days and plates were incubated at 37°C and 5% CO_2_. Photographs of wound closure were taken using a Motic AE31E microscope with Moticam BTU8 Tablet (40× magnification) at 24 h intervals for 3 days and analyzed using ‘ImageJ’ image processing program (NIH, Rockville, MD, USA). *In-vitro* invasion assay was performed for PC-3 and DU-145 cells as described in Ratnayake, *et al*. using BME (0.2×) as the inner coating of the transwell plates [20]. Cells were treated with *si*RNA as described above and plates were incubated at 37°C and 5% CO_2_ for 3 days. Crystal violet (0.5%) was used to stain the cells adhered to the bottom surface of the membrane in transwell inserts for the visualization and analysis as described in Ratnayake, *et al*. [20].

### Immunofluorescence microscopy

Slides were prepared for PC-3 and DU-145 cells as previously described in Ratnayake, *et al.* [20]. for aPKC specific *si*RNA treatments. At the end of the 2^nd^ day of treatment, cells were stained for either PKC-ι or PKC-ζ (red, 1:1000; A32727, anti-mouse secondary antibody) and Vimentin (green, 1:1000; A21206, anti-rabbit secondary antibody) to visualize under the Leica DM2000 upright fluorescent microscope using a Spot Flex cooled CCD Camera (Leica Microsystems Inc., Buffalo Grove, IL) (63X magnification). Images were captured and analyzed using ‘Spot Basic’ software (SPOT Imaging, Sterling Heights, MI). DAPI was used to visualize the nucleus (S36938; blue) [20].

### Immuno-gold transmission electron microscopy

PC-3 and DU-145 were seeded in six well plates (4 × 10^4^ cells/well). After 24 h post seeding time *si*RNA of PKC-ι or PKC-ζ (20 nM) treatments were conducted against scrambled *si*RNA for two days. Cell pellets were collected at the end of 48 h incubation period and processed as described previously by Wilkens, *et al*. [45]. Grids made from fixed and embedded cells were blocked with 1% BSA in PBS after washing with PBS, 20 mM glycine in PBS. Then the grid were incubated with either primary antibodies of PKC-ι or PKC-ζ (mouse monoclonal antibodies, 1:80) for 1 h at room temperature followed with secondary antibody-gold-complexes (20 nm, goat anti-mouse- ab27242, 1:50). Grids were then incubated with Vimentin primary antibody (rabbit monoclonal antibody, 1:80) followed by secondary antibody-gold-complexes (10 nm, goat anti-rabbit- ab27234, 1:50). Clean grids were dried overnight in a desiccator and analyzed using transmission electron microscopy (FEI Morgagni, Eindhoven, Netherlands) with a 16.7 Mega Pixel bottom mount camera.

### Knockdown of expression PKC-ι, PKC-ζ, PRRX1 and SNAIL1

PC-3 and DU-145 (1 × 10^5^) cells were seeded in T25 flasks and after 24 h post seeding time, fresh medium was supplied and *si*RNA (20 nM for PKC-ι/ζ or 30 nM for SNAIL1 or PRRX1) treatments were conducted against scrambled *si*RNA (control) for 48 h using ‘siTran’ *si*RNA transfection reagent (TT300002, Origene Technologies, Inc.) according to the manufacturer’s recommended ratios. The cell pellets were collected at the end of 48 h incubation period to perform Western blot experiments or qPCR experiments as described in Win, *et al.* [7]. Duplex sequences used in *si*RNAs are as follows. PKC-ι; rGrUrArUrUrCrArCrUrUrCrArArArUrCrArUrArArArCrUTA, PKC-ζ; rGrArGrGrArArUrArArArArUrGrUrUrCrCrGrArUrGrUrUGT, and SNAIL1; NM_005985 and PRRX1; NM_006902.4.

### Quantitative real-time PCR (qPCR)

RNA was isolated from *si*RNA treated PC-3 and DU-145 cell lysates qPCR was performed as previously described in Ratnayake, *et al*. [46]. Following primers were used. PKC-ι; forward: TTGCAATGAGGTTCGAGACA; reverse: CTGAGATGATACTGTACACGGG. PKC-ζ; forward: ACCCCTTCCTGGTCGGATTA; reverse: AGGGGGCTTCTGGAAGAGTA. Vimentin; forward ACACCCTGCAATCTTTCAGACA; reverse GATTCCACTTTGCGTTCAAGGT. E-cadherin; forward: AGGCCAAGCAGCAGTACATT, reverse: ATTCACATCCAGCACATCCA. SNAIL1; forward: CGAGTGGTTCTTCTGCGCTA; reverse: TGCAGCTCGCTGTAGTTAGG. PRRX1; forward: CCCACATCTCTTTCGTGCCT; reverse: GAACAGTGGTGAGGGTGTGT. β-actin; forward: AGAGCTACGAGCTGCCTGAC and reverse, AGCACTGTGTTGGCGTACAG was used as the housekeeping gene.

### Western blot analysis and densitometry

The detailed procedure of the Western blots was performed as termed in Ratnayake, *et al*. [46]. Western blot band intensities were calculated using ‘AlphaView’ software (ProteinSimple Inc., San Jose, CA, USA) in which the baseline intensity was subtracted from each band’s intensity to obtain the protein’s corrected intensity.

### Xenografts

Prostate carcinoma cells (PC-3 and DU-145) were seeded, grown and harvested to conduct subcutaneous injections with 1 × 10^6^ cells into the flank of athymic nude mice, which were 7 weeks old. Twenty male mice were included in a group and four groups were created as treated and untreated for each aPKC inhibitor treatment (ICA-1 T and ζ-Stat). Treatment groups were given ICA-1 T (80 mg/kg) and ζ-Stat (40 mg/kg) against control groups separately by subcutaneous injections every day from the starting point where a tumor reached the diameter of 0.2 cm. 80 mg/kg for ICA-1 T and 40 mg/kg for ζ-Stat were selected based on preliminary results carried out for 20–200 mg/kg concentration range where c-reactive protein (CRP), aminotransferase (AST), gamma-glutamyl transpeptidase (GGT), and Troponin I levels showed no significant difference compared to the control. Sodium Chloride 0.9% (Normal Saline, Cat. Number, Z1376, Fisher Scientific, Inc.) was used as control. Tumor sizes were recorded daily using an external digital caliper. Tumors were excised when the end points were reached (when a tumor from any experimental condition reached the size of 2 cm). All animal experimental procedures were reviewed and approved by the Institutional Animal Care and Use Committee of University of South Florida, Tampa, FL, USA (IACUC number IS00005822) in accordance with the US NIH Guidelines for animal research.

### Immunohistochemistry

Immunohistochemistry (IHC) was performed for the xenografts extracted for ICA-1 T treatments. For each sample, blocking was performed following deparaffinization of the sections, and citrate microwave antigen retrieval. PKC-α was detected after incubated with rabbit monoclonal anti-PKC-α (1:100 dilution; ab32376, Abcam) for 60 minutes. ‘EnVision’ detection system was used. Separate sections were subsequently incubated over night with purified rabbit polyclonal anti-PKC-ι (1:100 dilution; ab5282, Abcam), rabbit polyclonal anti-PKC-ζ (1:100 dilution; ab59364, Abcam) and rabbit monoclonal anti-Vimentin (5741S, Cell Signaling Technology). This research was assisted in part by the Analytic Microscopy Core Facility at the H. Lee Moffitt Cancer Center & Research Institute, an NCI designated Comprehensive Cancer Center (P30-CA076292).

### Statistical treatment

All data shall be seen as mean ± SD. The statistical study was carried out with one or two-way ANOVA followed by the Tukey HSD test as multiple comparisons tests using the statistical research online tool ‘VassarStats’ (http://vassarstats.net/anova1u.html). P-value <0.05 or <0.01 shown statistical significance.

## Results

### aPKC signaling contributes to prostate cancer cellular metastasis

**Figure 1.**
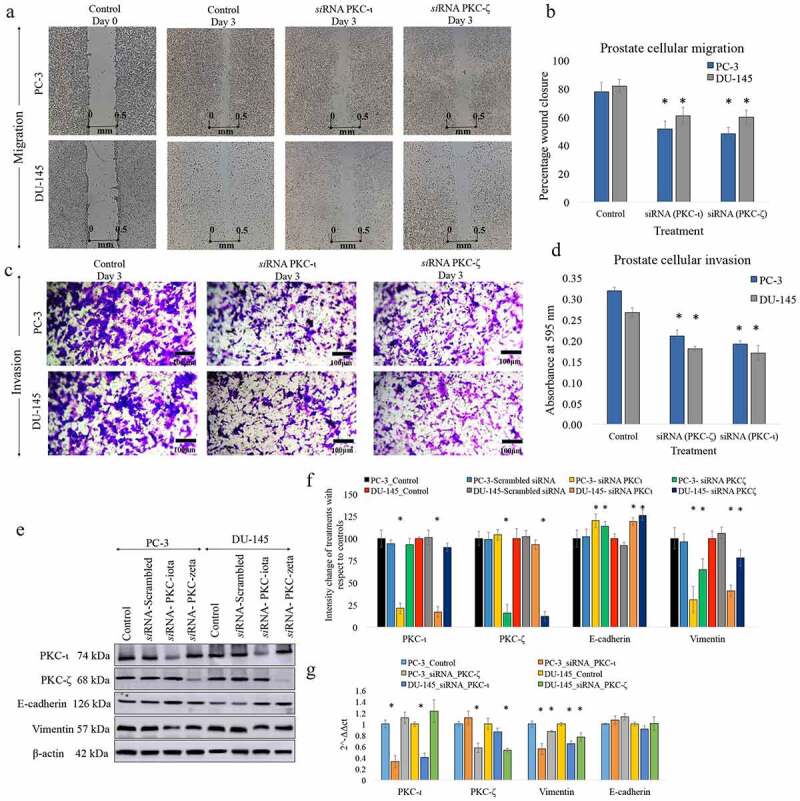
aPKC knockdown decreases prostate cancer cell migration and invasion. Figure 1(a) and 1(b) represent the effects of aPKC *si*RNA treatments (20 nM against scrambled *si*RNA) on PC-3 and DU-145 cell migration in wound healing assay and Figure 1c and 1d represent the effects of aPKC *si*RNA treatments on prostate cell invasion in Boyden chamber assay in the presence of basement extract (BME). In the wound healing assay, microscopic photographs (40×) of scratches on cells at the beginning (day 0) were compared with the photographs taken after 3 days. The effects of aPKC attenuation are shown compared to their controls. Experiments were performed for each cell line and representative photographs are shown (*N* = 3). Figure 1b bar graph represents a comparison of calculated percent wound closure for the photographs taken using ImageJ (NIH, Rockville, MD, USA). For the Boyden chamber assay (Figure 1b), invaded cells in the bottom surface of transwell insert were stained with 0.5% crystal violet and microscopic photographs were taken (100×). Subsequently, crystal violet was dissolved in 70% ethanol and absorbance was measured at 590 nm which is directly proportional to the number of invaded cells (Figure 1d). Figure 1e shows the effect of RNA interference (*si*RNA) of PKC-ι and PKC-ζ in prostate cancer cells (PC-3 and DU-145). PC-3 and DU-145 (1 × 10^5^) cells were seeded in T25 flasks and after 24 h post seeding time, fresh medium was supplied and *si*RNA (20 nM for PKC-ι/ζ or 30 nM for SNAIL1 or PRRX1) treatments were conducted for 48 h using ‘siTran’ *si*RNA transfection reagent (TT300002, Origene Technologies, Inc.) according to the manufacturer’s recommended ratios. The expression of the protein levels of PKC-ι, PKC-ζ, E-cadherin and Vimentin are shown. Total protein (80 μg) was loaded into each well and β-actin was used as the internal control in each Western blot. Experiments were performed in each trial and representative bands are shown (*N* = 4). The blots are cropped from different gels and separated with a white space between them. Densitometry values for the Western blots are also shown (figure 1(f)). Figure 1(g) shows the mRNA levels of PKC-ι, PKC-ζ, E-cadherin and Vimentin for aPKC attenuation for respective samples based on quantitative real-time PCR (qPCR) (*N* = 3). All values are reported as the means ± SD. Statistical significance is indicated by an asterisk (*P≤<0.05)

The effects of knockdown of PKC-ζ and PKC-ι expression using *si*RNA and selective inhibition of PKC-ι and PKC-ζ using specific inhibitors for prostate cancer cell migration/invasion were first determined. The half-maximal inhibitory concentration (IC_50_) of two novel inhibitors ICA-1 T (2.5 μM), a specific PKC-ι inhibitor and ζ-Stat (5 μM), a PKC-ζ inhibitor were compared with PKC-ζ and PKC-ι *si*RNA (20 nM) treatments. Wound-healing assay was executed to examine the outcome of aPKC attenuation on *in-vitro* prostate cancer cellular migration. Based on preliminary results, we found that knockdown of expression of aPKCs using *si*RNA and specific inhibition through specific inhibitors demonstrated same pattern but *si*RNA treatment were emerged as the more efficient method of diminution of aPKCs. Therefore *si*RNA method was selected for the later experiments *in-vitro*. As demonstrated in Figure 1(a), comparisons were made of the snapshots of each cell as ‘day 0’ (initial point) and ‘day 3’ (end point). In addition, the wound (scratch) areas were estimated and attributed to the relevant controls in order to evaluate the statistical validity of wound closure (Figure 1(b)). Compared to 78% wound closure in control sample of PC-3, *si*RNA treatments demonstrated less wound closures as 51% (*P* < 0.05) for *si*RNA of PKC-ι and 48% (*P* < 0.05) for *si*RNA of PKC-ζ. In comparison to 82% wound closure in DU-145 control, 61% (*P* < 0.05) for *si*RNA of PKC-ι and 60% (*P* < 0.05) for *si*RNA of PKC-ζ (Figure 1(a) and Figure 1(b)) were obtained. These results suggested that *si*RNA treated samples for aPKCs decreased the cellular migration by 34% (*P* < 0.05) and 26% (*P* < 0.05) for PC-3 and DU-145 cell lines, respectively for *si*RNA of PKC-ι treatments. Similarly 38% (*P* < 0.05) and 27% (*P* < 0.05) inhibition of migration was obtained PC-3 and DU-145 cell lines, respectively for *si*RNA of PKC-ζ (Figure 1(a) and Figure 1(b)).

Moreover, basement membrane extract (BME) invasion assay was executed to examine the outcome of aPKC attenuation on *in-vitro* prostate cancer cellular invasion. Invaded cells were treated with crystal violet on the transwell inserts and snapshots were captured as the visual representation of the invasion assay in randomly selected fields (Figure 1(c)). Crystal violet stained cells were then dissolved into the lower chamber in 70% ethanol and the absorbency was determined at 590 nm, which is directly proportional to the degree of invaded cells. (Figure 1(d)). These results suggested that *si*RNA treated samples for aPKCs decreased the cellular invasion by 38% (*P* < 0.05) and 33% (*P* < 0.05) for PC-3 and DU-145 cell lines, respectively, for *si*RNA of PKC-ι treatments. Similarly 32% (*P* < 0.05) and 29% (*P* < 0.05) inhibition of cellular invasion was obtained for PC-3 and DU-145 cell lines, respectively, for *si*RNA of PKC-ζ treatments (Figure 1(c) and Figure 1(d)).

Seeking more specifically, we determined the efficiencies of *si*RNA knockdown of expression for both aPKCs along with the effects of aPKC attenuation on Vimentin and E-cadherin, a mesenchymal and an epithelial marker, respectively. As demonstrated in Figure 1(e), immunoblots shown that aPKC specific *si*RNA treatments for PC-3 and DU-145 cell lines significantly reduced the PKC-ι and PKC-ζ protein expression. *si*RNA for PKC-ι decreased the total PKC-ι levels by 79% (*P* < 0.05) and 83% (*P* < 0.05) without having a significant effect on PKC-ζ expression for PC-3 and DU-145 cell lines, respectively (Figure 1(e) and Figure 1(f)). Similarly, *si*RNA for PKC-ζ decreased the PKC-ζ levels by 84% (*P* < 0.05) and 88% (*P* < 0.05) without having a significant effect on PKC-ι expression for PC-3 and DU-145 cells, respectively (Figure 1(e) and Figure 1(f)). These results confirmed the high specificity and the efficiency of the experimented *si*RNA conditions. As demonstrated in Figure 1(e) and Figure 1(f), Vimentin expression was diminished by 69% (*P* < 0.05) and 59% (*P* < 0.05) for PKC-ι knocked-down of PC-3 and DU-145 samples, respectively, while PKC-ζ knockdown resulted a diminution of Vimentin expression by 35% (*P* < 0.05) and 22% (*P* < 0.05) for PC-3 and DU-145 cells, respectively. Interestingly, E-cadherin expression was elevated by 20% (*P* < 0.05) and 19% (*P* < 0.05) for PKC-ι knocked-down PC-3 and DU-145 samples, respectively, while PKC-ζ knockdown resulted an upregulation of E-cadherin expression by 14% (*P* < 0.05) and 26% (*P* < 0.05) for PC-3 and DU-145 cells, respectively. We have also analyzed the mRNA levels of aPKCs, Vimentin and E-cadherin upon aPKC attenuation (Figure 1(g)). Both aPKC m RNA levels decreased significantly (*P* < 0.05) for the respective *si*RNA treatment without having an effect on the other aPKC member. mRNA levels of Vimentin were also reduced expressively (*P* < 0.05) for the both aPKC attenuations as observed in Western blots. Interestingly, E-cadherin mRNA levels did not show a significant alteration as a result of aPKC diminution but Western blots indicated that E-cadherin protein levels increased as a result of aPKC diminution and which suggests that E-cadherin degradation reduced and stabilizing the remaining E-cadherin levels. These results suggest that both aPKCs play an active role in the upregulation of prostate cancer cell motility possibly via accelerating EMT of the prostate tumor cells which was indicated by the alterations of E-cadherin and Vimentin levels upon aPKC reduction.

### Diminution of aPKC expression downregulates EMT signaling of prostate cancer cells

**Figure 2.**
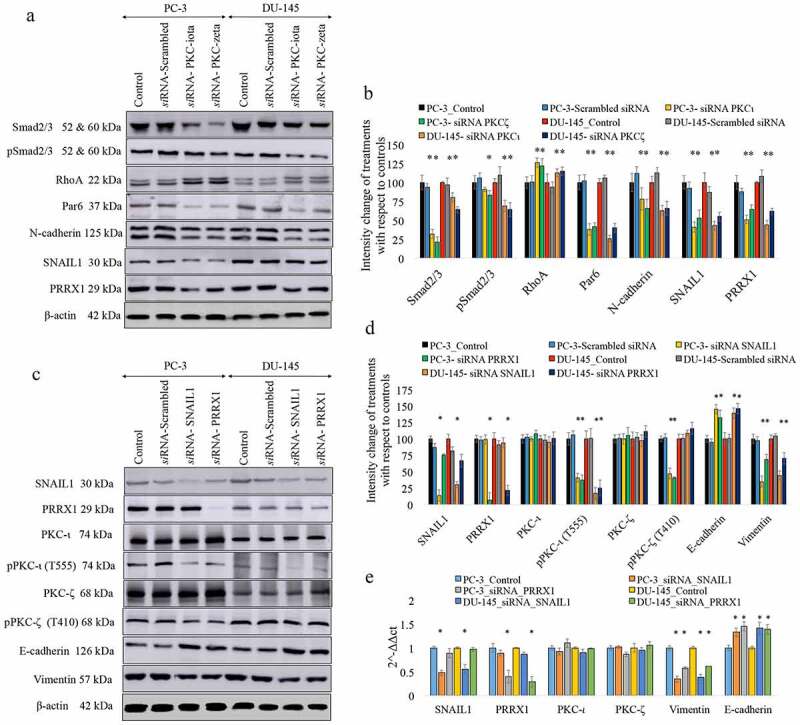
Effects of RNA interference (*si*RNA) for aPKCs, transcription factors of PRRX1 and SNAIL1 on the expression of EMT markers in prostate cancer cells. Figure 2(a) and 2(b) show the expression of the protein levels for EMT markers for aPKC attenuation (Smad2/3, pSmad2/3 RhoA, Par6 and N-cadherin) and the transcription factors of SNAIL1 and PRRX1. Figure 2c and 2d show the expression of SNAIL1, PRRX1, total PKC-ι, phosphorylated PKC-ι (T555), total PKC-ζ, phosphorylated PKC-ζ (S410), E-cadherin and Vimentin for the *si*RNA knockdown of the expression of SNAIL1 for PC-3 and DU-145 cell lines. PC-3 and DU-145 (1 × 10^5^) cells were seeded in T25 flasks and after 24 h post seeding time, fresh medium was supplied and *si*RNA (20 nM for PKC-ι/ζ or 30 nM for SNAIL1 or PRRX1) treatments were conducted for 48 h using ‘siTran’ *si*RNA transfection reagent. Total protein (80 μg) was loaded into each well and β-actin was used as the internal control in each Western blot. Representative densitometry values for the Western blots are shown (Figure 2(b) and 2(d)). Experiments were performed and representative bands are shown (*N* = 4). The blots are cropped from different gels and separated with a white space between them Fig. 2E shows the mRNA levels of SNAIL1, PRRX1, PKC-ι, PKC-ζ, E-cadherin and Vimentin for SNAIL1 and PRRX1 *si*RNA knockdown samples for PC-3 and DU-145 cells based on quantitative real-time PCR (qPCR) (*N* = 3). All values are reported as the means ± SD. Statistical significance is indicated by an asterisk (*P < 0.05)

Next, we examined in more detail EMT giving emphasis to the proteins such as Smad2/3, pSmad2/3, RhoA, Par6 and N-cadherin along with the transcription factors SNAIL1 and PRRX1 using Western blots. Our previous reports confirmed that Smad2/3 and Par6/aPKC/RhoA pathways upregulate the expression of Vimentin along with aPKCs in melanoma cells [20]. The same trend was detected in PC-3 and DU-145 (Figure 2(a) and Figure 2(b)). Expression of Par6 and N-cadherin, total Smad2/3 along with the phosphorylated Smad2/3 significantly decreased (*P* ≤ 0.05) as a result of both aPKC *si*RNA treatments (Figure 2(a) and Figure 2(b)). Reduction of pSmad2/3 indicates that the activated Smad2/3 is downregulated as a result of aPKC attenuation. Taken together with the reduction of Vimentin expression and upregulation of E-cadherin, these results further confirmed the diminution of EMT in prostate cancer cells. Supplementary Figure 1a-1d demonstrates a summary of *in-vitro* effects of ICA-1 T (2.5 μM) and ζ-Stat (5 μM) on the migration and invasion assays for metastatic PC-3 and DU-145 cell lines. Supplementary Figure 1e-1f show the Western blot analysis of the markers that we discussed in Figures 1(e) and Figure 2(a). Here, we have compared the effects aPKC attenuation in metastatic prostate cancer cell lines PC-3 and DU-145 against non-metastatic RWPE-1 normal prostate cell line. Results confirmed that the overall outcome of specific inhibition of aPKCs are very similar to *si*RNA treatments on the two metastatic cell lines but inhibitors provided lesser efficiency compared to *si*RNA applications. However, non-metastatic RWPE-1 cells did not demonstrate a significant alteration of protein levels for the tested markers indicating normal prostate cells are not dependent on aPKCs. But metastatic cell lines demonstrated a significant dependency on aPKCs in their progression. Based on these preliminary results, we decided to analyze the role of aPKCs on metastatic cells using aPKC *si*RNA to pinpoint the downstream effects of aPKC diminution.

Protein levels of SNAIL1 and PRRX1 changed significantly as a result of aPKC *si*RNA knockdown (Figure 2(a) and Figure 2(b)). In the preliminary stage we have tested more transcription factors connected to Vimentin expression such as ZEB1, TWIST and SNAIL2 along with SNAIL1 and PRRX1. These TFs have highly specific expression profiles and known to upregulate Vimentin expression during EMT [39–44]. Only SNAIL1 and PRRX1 expressions were significantly changed as a result of *si*RNA knockdown of both aPKCs (Figure 2(a) and Figure 2(b)). Subsequently, We’ve checked the impact of *si*RNA knockdown of the expression of PRRX1 and SNAIL1 on the expression SNAIL1, PRRX1, PKC-ι, phospho-PKC-ι (T555), PKC-ζ, phospho-PKC-ζ (T410), E-cadherin and Vimentin (Figure 2(c) and Figure 2(d)). Western blots of SNAIL1 and PRRX1 demonstrated a significant decrease (*P* < 0.05) of the protein expression upon their respective *si*RNA treatments which confirmed the specificities of *si*RNA over targeted transcription factors (Figure 2(c) and Figure 2(d)). E-cadherin expression was significantly increased while Vimentin expression was significantly decreased as a result of diminution of both SNAIL1 and PRRX1. Both aPKC levels did not alter significantly. Interestingly, phospho-PKC-ι (T555) levels decreased markedly for both cell lines upon *si*RNA knockdown of SNAIL1 and PRRX1 while phospho-PKC-ζ (T410) level decreased significantly only in PC-3 cell line for both *si*RNA knockdown of SNAIL1 and PRRX1 (Figure 2(c) and Figure 2(d)). T555 locates in the ‘turn motif’ of PKC-ι structure while T410 locates on ‘activation loop’ of PKC-ζ structure and phosphorylation in these locations are required for the complete maturation and activation of aPKCs [47]. Therefore degree of phosphorylation can be observed through these sites which indicate activities. SNAIL1 and PRRX1 downregulation lead to lower the activities of both aPKCs which negatively affects Vimentin dynamics. mRNA levels of SNAIL1, PRRX1, Vimentin, E-cadherin (Figure 2(e)) and aPKCs to validate and confirm the Western blot results.

### Vimentin is a novel PKC-ι and PKC-ζ binding partner

As a result of the close relationship we observed between aPKCs and Vimentin, we focused our investigations on the interaction of aPKCs with Vimentin. As demonstrates in the Supplementary Figure 2, preliminary results of immunoprecipitation/Western blot (IP/WB) and reverse-IP/WB for Vimentin, PKC-ι, PKC-ζ, results indicated a strong association of Vimentin with both aPKCs.

**Figure 3.**
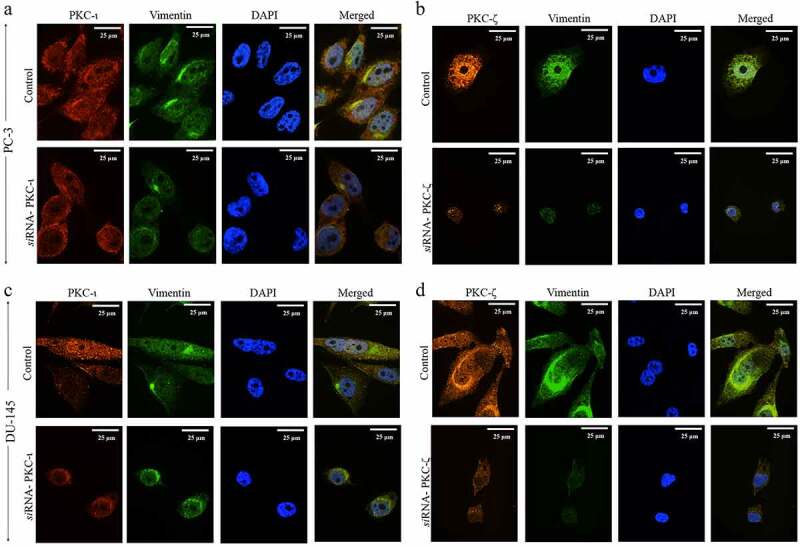
PKC-ι and PKC-ζ associate with Vimentin and regulate Vimentin dynamics in prostate cancer cells. PC-3 and DU-145 (2.5 × 10^3^) cells were seeded in chamber slides and after 24 h post seeding time, fresh medium was supplied and *si*RNA (20 nM for PKC-ι/ζ) treatments were conducted for 48 h using ‘siTran’ *si*RNA transfection reagent. Slides were prepared as explained in the methods section. Fig. 3A represents the immunofluorescence staining of PKC-ι (red panel) and Vimentin (green panel), nuclei (blue panel) along with the merged image for PC-3 cells treated with PKC-ι *si*RNA against its control. Fig. 3B represents the immunofluorescence staining of PKC-ζ (red panel) and Vimentin (green panel), nuclei (blue panel) along with the merged image for PC-3 cells treated with PKC-ζ *si*RNA against its control. Similarly, Fig. 3 C indicates the immunofluorescence staining of PKC-ι (red panel) and Vimentin (green panel), nuclei (blue panel) along with the merged image for DU-145 cells treated with PKC-ι *si*RNA against its control. Fig. 3D represents the immunofluorescence staining of PKC-ζ (red panel) and Vimentin (green panel), nuclei (blue panel) along with the merged image for DU-145 cells treated with PKC-ζ *si*RNA against its control. The images were captured at 630X magnification. Experiments (*N* = 3) were performed

Figure 3 displays that immunofluorescence (IF) staining, which again confirmed the association of aPKCs and Vimentin. IF images of PC-3 cell line for Vimentin and PKC-ι and (Figure 3(a)) demonstrated both proteins spread throughout the cytoplasm but PKC-ι is concentrated along the plasma membrane (Figure 3(a)- red panel). Vimentin (Figure 3(a)- green panel) also concentrated along with PKC-ι. Both protein levels significantly decreased (lighter in staining) upon treatment of *si*RNA of PKC-ι (Figure 3(a) bottom panel). On the other hand, DU-145 cell line did not demonstrate any concentrated areas of the PKC-ι. Similar to PC-3, both protein levels significantly decreased (lighter in staining) upon treatment of *si*RNA of PKC-ι which again confirm the association of PKC-ι with Vimentin and the expression of Vimentin connected to aPKCs (Figure 3(c)- red and green panels). The same relationship was observed between PKC-ζ and Vimentin based on the IF images for both PC-3 cells (Figure 3(b)) and DU-145 cells (Figure 3(d)). In PC-3 cells tested for PKC-ζ and Vimentin (Figure 3(b)- red and green panels) demonstrated that both proteins distributed throughout the cytoplasm but PKC-ζ (Figure 3(b)- red panel) does not show any concentrated regions like PKC-ι does close to the plasma membrane (Figure 3(a)- red panel). Both protein levels significantly decreased (lighter in staining) upon treatment of *si*RNA of PKC-ζ in PC-3 cells (Figure 3(b)- bottom panel). The same trend was detected for DU-145 cells in Vimentin and PKC-ζ images (Figure 3(d)- red and green panels). In addition, IF images for *si*RNA treatments for both cell lines demonstrated other characteristics such as nuclei shrinkage and overall cell size reduction of cells (Figure 3(a)-Figure 3(d)- bottom panels). In addition, controls samples of both cells (Figure 3(a)-Figure 3(d)- top panels), clearly demonstrate the invasive features such as development of invadopodia, filopodia and lamellipodia. However aPKC attenuated samples (Figure 3(a)-Figure 3(d)- bottom panels) showed less of such features. Overall we demonstrated lesser cells in the *si*RNA treated samples with unhealthy conditions with respect to their controls.

**Figure 4.**
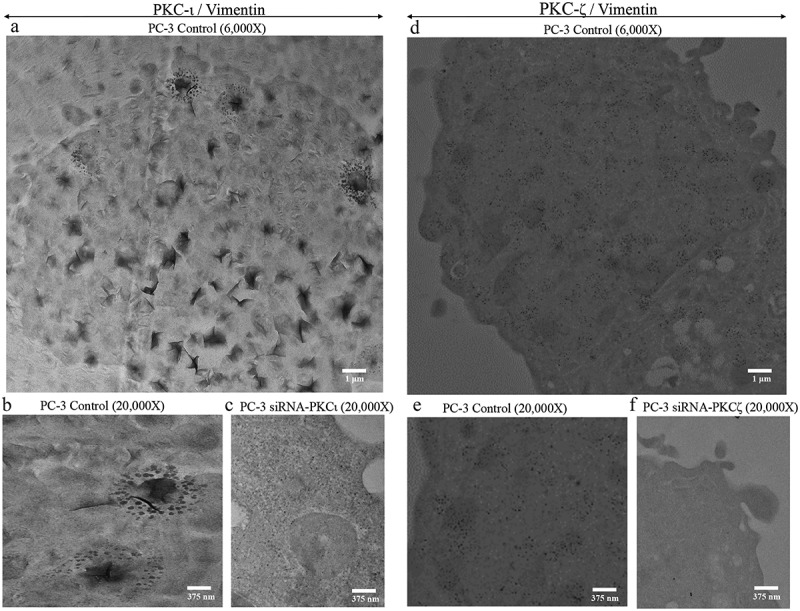
Immuno-gold transmission electron microscopic (IG-TEM) images of PKC-ι with Vimentin in PC-3 cells. PC-3 and DU-145 were seeded in six well plates (4 × 10^4^ cells/well). After 24 h post seeding time *si*RNA of PKC-ι or PKC-ζ (20 nM) treatments were conducted against scrambled *si*RNA for two days. Cells were prepared as explained in the methods. Fig. 4A shows a 6000X magnified PC-3 cell where PKC-ι was tagged with 20 nm gold particles and Vimentin was tagged with 10 nm gold particles. Fig. 4B showed an enlarged area (20,000X) of the same cell displayed in Fig. 4A. Fig. 4 C demonstrates an enlarged area of a PC-3 cell which was treated with PKC-ι *si*RNA which indicates PKC-ι concentrated, areas disappeared as a result of *si*RNA treatments. Fig. 4D (6000X) and 4E (20,000X) showed no concentrated areas of PKC-ζ or Vimentin but both proteins heavily distributed throughout the cytosol in PC-3 cells. Fig. 4 F shows an enlarged area of a PKC-ζ *si*RNA treated PC-3 cell which shows lesser PKC-ζ and Vimentin compared to its control

Since we observed PKC-ι being concentrated along the plasma membrane of PC-3 cell lines in IF images (top red panel of Figure 3(a)), which was different to the distribution of PKC-ι and PKC-ζ in DU-145 (also, PKC-ζ distribution in PC-3), we have decided to further investigate the distribution of PKC-ι with Vimentin based on immuno-gold transmission electron microscopy (IG-TEM) (Figure 4). We used 20 nm gold particles to tag PKC-ι and PKC-ζ and 10 nm gold particles to tag Vimentin in separate experiments. Figure 4(a) (6000X) shows a PC-3 cell in the control sample which clearly shows PKC-ι concentrated areas close to the plasma membrane. Figure 4(b) (20,000X) displayed an enlarged area of the same cell which shows 20 nm gold particles which represents PKC-ι clustered along with 10 nm particles which represent Vimentin. Figure 4(c) (20,000X) demonstrates an enlarged area of a PC-3 cell which was treated with *si*RNA for PKC-ι. This picture shows low density of both 20 nm and 10 nm particles indicating lower expression of their respective tagged proteins; PKC-ι and Vimentin. Also concentrated areas of PKC-ι and Vimentin were not observed upon PKC-ι *si*RNA treatments. These results indicate that PKC-ι tend to concentrate along the membrane of PC-3 cells and distribution significantly reduced with *si*RNA of PKC-ι. Interestingly, Vimentin expression in PC-3 cells also significantly reduced upon PKC-ι diminution. These results (Figure 4(a)-Figure 4(c)) agreed with the IF images obtained for PC-3 cells for PKC-ι and Vimentin (Figure 3(a)). Similarly, PC-3 cells were tested for PKC-ζ (20 nm tagged nanoparticles) along with Vimentin (10 nm tagged particles). As shown in Figure 4(d) (6000X) and 4E (20,000X), no specific concentrated areas of PKC-ζ were observed for PC-3 control samples compared to PKC-ι controls (Figure 4(a)), but have shown abundant levels of PKC-ζ and Vimentin throughout the cell. PKC-ζ *si*RNA treatments significantly decreased the abundancy of both nanoparticles which indicates lower levels of their respective proteins (PKC-ζ and Vimentin) (figure 4(f)). These results again indicated PKC-ζ also strongly associates with Vimentin and Vimentin levels decreased as a result of PKC-ζ attenuation (figure 4(f)).

### Vimentin is a novel aPKC kinase target

**Figure 5.**
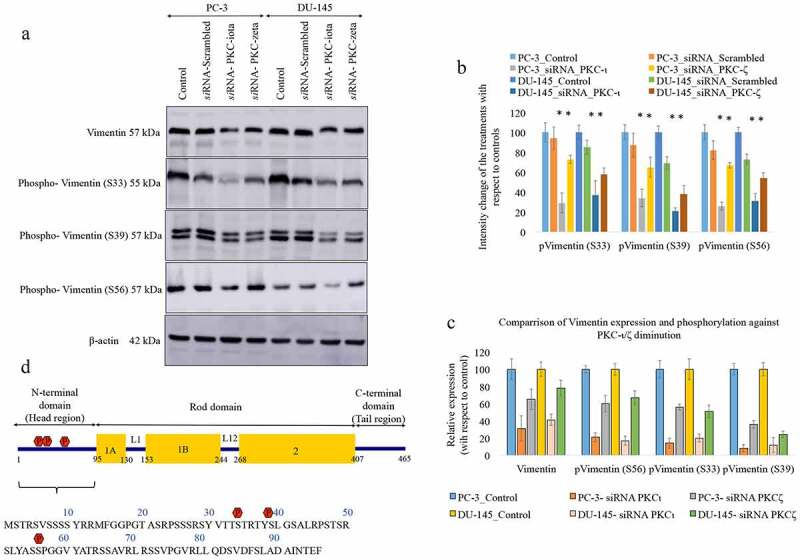
Phosphorylation of Vimentin at Ser33, Ser39 and Ser56 decreases due to aPKC attenuation. Fig. 5A shows that the degree of phosphorylation at Ser33, Ser39 and Ser56 significantly decreased as a result of *si*RNA of both PKC-ι/ζ. Fig. 5A shows the effects of *si*RNA of PKC-ι and PKC-ζ in prostate cancer cells (PC-3 and DU-145) and Fig. 5B represents the densitometry values for the Western blots. Total protein (80 μg) was loaded into each well and β-actin was used as the internal control in each Western blot. Densitometry values for the Western blots are also shown. Experiments (*N* = 4) were performed in each trial and representative bands are shown. The blots are cropped from different gels and separated with a white space between them. Densitometry values are reported as the means ± SD. Statistical significance is indicated by an asterisk (*P ≤ 0.05). Fig. 5 C demonstrates a comparison of the degree of reduction of total Vimentin compared to the reduction of phosphorylation at Ser33, Ser39 and Ser56 sites on Vimentin for aPKC attenuation. Fig. 5D demonstrates an animation of the Vimentin structure where aPKC targeted phosphorylation sites in the head region of Vimentin structure are marked in red color

So far our results indicated that Vimentin tends to bind with both aPKCs. In addition, the protein levels of two of the most important transcription factors (PRRX1 and SNAIL1) that govern Vimentin expression, decreased as a result of aPKC diminution (Figure 2(a) and Figure 2(b)). Furthermore, down-regulation of PRRX1 and SNAIL1 negatively affected the activated aPKC levels which were confirmed by the reduction of phospho-PKC-ι (T555) and phospho-PKC-ζ (T410) without affecting their total protein levels (Figure 2(c) and Figure 2(d)). Then, we tested whether aPKCs involved in the Vimentin dynamics by participating the phosphorylation events on Vimentin. We have tested the degree of phosphorylation of the critical phosphorylation sites on Vimentin structure such as Ser6, Ser33, Ser39, Ser56 and Ser71 upon aPKC *si*RNA knockdown. Out of these sites we found that Ser33, Ser39 and Ser56 are heavily affected as a result of both aPKC *si*RNA knockdown of expression. As Figure 5(a) and Figure 5(b) indicate, the degree of phosphorylation significantly decreased at Ser33, Ser56 and Ser56 for both cell lines upon *si*RNA treatments of PKC-ι and PKC-ζ. Figure 5(c) demonstrates a comparison of the degree of reduction of total Vimentin compared to the reduction of phosphorylation at Ser33, Ser39 and Ser56 sites on Vimentin for aPKC attenuation. Results indicated that all three phosphorylation sites demonstrated a greater degree of reduction of phosphorylation than the reduction of total Vimentin as a result of aPKC attenuation in the order of Ser56, Ser33 and Ser39, in which Ser39 being the highest for both aPKCs. Based on the significance, PKC-ι serves as the major kinase with respect to PKC-ζ. Results indicate that VIF dynamics are retarded when both aPKCs are attenuated. Figure 5(d) exhibits the Vimentin structure where aPKC targeted phosphorylation sites in the head region are marked in red color.

**Figure 6.**
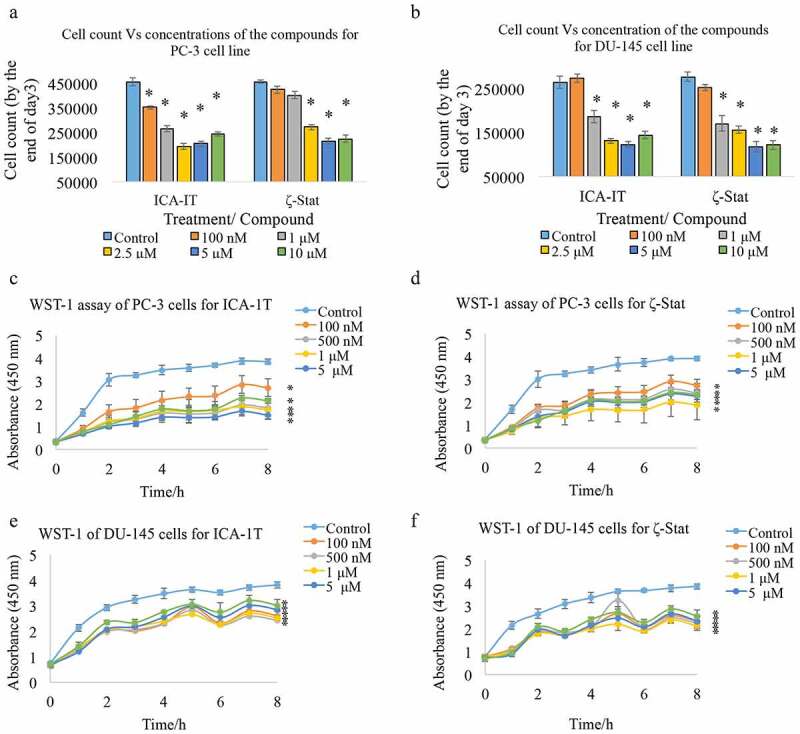
ICA-1 T and ζ-Stat are effective aPKC specific inhibitors *in-vitro*. Fig. 6A and 6B demonstrate the *in-vitro* effects of aPKC inhibitors (ICA-1 T and ζ-stat) on cell proliferation, cell viability and cytotoxicity for PC-3 and DU-145 prostate cancer cells, respectively. Approximately 1 × 10^5^ were cultured in T75 flasks and treated with either equal volume of sterile water (control) or inhibitors (0.10–10 µM). Additional doses of sterile water or inhibitors were supplied every 24 h during a 3 day incubation period. Subsequently, cells were lifted and counted. As shown in Fig. 6 C-6 F, cytotoxicity of aPKC inhibitors was measured using WST-1 assay for PC-3 and DU-145 cell lines, respectively. The absorbance at 450 nm is due to production of water soluble formazan and was measured as a function of time. The absorbance is directly proportional to the number of cells. Experimental concentrations used in WST-1 assay for ICA-1 T and ζ-Stat were the same concentrations used for the dose curve. The absorbance at 450 nm against time is plotted. Experiments (*N* = 3) were performed for each cell line and mean ± SD are plotted. Statistical significance is indicated by asterisk (*P < 0.05)

**Figure 7.**
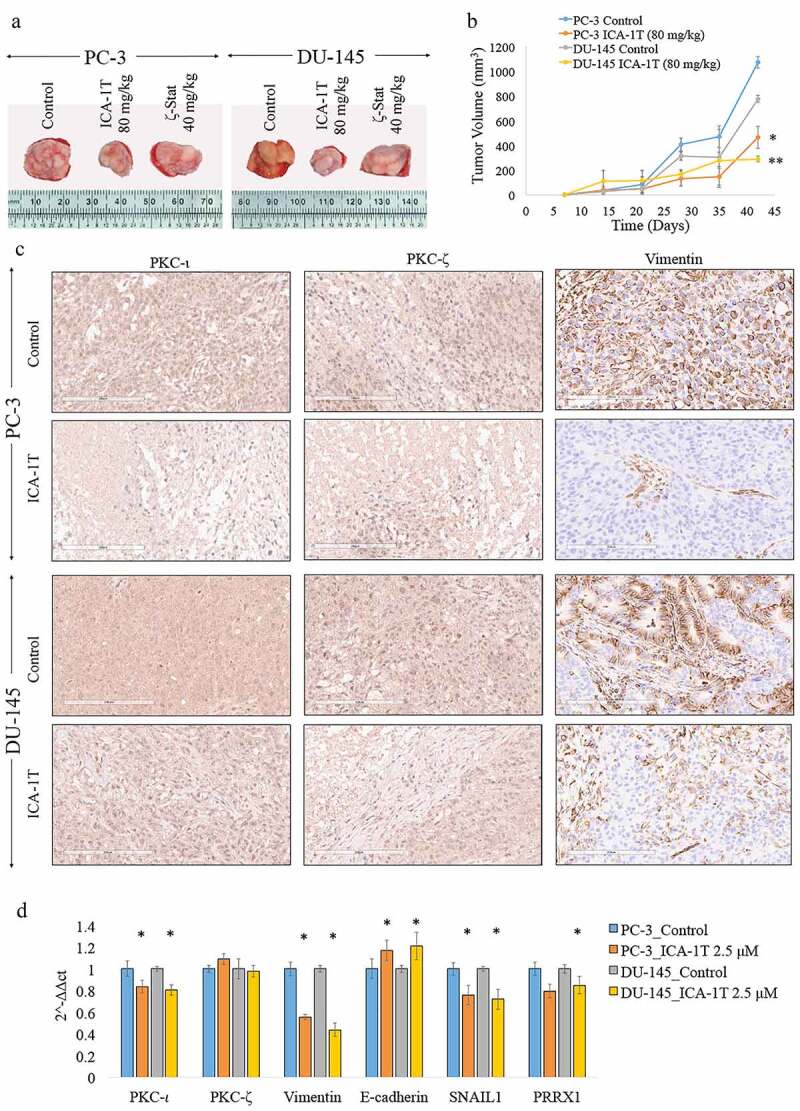
*In-vivo* attenuation of aPKCs using PKC-ι specific ICA-1 T and PKC-ζ specific ζ-Stat inhibitor compounds. Fig. 7A shows representative photographs of the tumors excised for both ICA-1 T (80 mg/kg) and ζ-Stat (40 mg/kg) treatments against their controls for PC-3 and DU-145 xenografts, respectively. Fig. 7B shows the tumor growth (volume) against the time (number of days) for ICA-1 T treatments with respect to their controls for PC-3 and DU-145 cells. Fig. 7 C indicates microscopic images of immunohistochemical staining of PKC-ι, PKC-ζ and Vimentin in PC-3 and DU-145 xenogrfts. Tumors were excised, processed and stained for above markers with PKC-α antibody as a control for PKC-ι and PKC-ζ staining. Left panel depicts PKC-ι staining, middle panel depicts PKC-ζ and right panel depicts Vimentin for PC-3 and DU-145 xenografts, respectively. Magnification for all micrographic images were 200X and representative images were shown. Fig. 7D shows the mRNA levels of PKC-ι, PKC-ζ, E-cadherin, Vimentin, SNAIL1 and PRRX1 for ICA-1 T treated subcutaneous tumors of PC-3 and DU-145 against respective control samples based on quantitative real-time PCR (qPCR) (*N* = 3). All values are reported as the means ± SD. Statistical significance is indicated by an asterisk (*P < 0.05)

We used two novel aPKC specific inhibitors, ICA-1 T (specific to PKC-ι) and ζ-Stat (specific to PKC-ζ) for the *in-vivo* experiments. As shown in Figure 6(a) and Figure 6(b), according to *in-vitro* cell population assay, ICA-1 T demonstrated 57.5% and 50.0% inhibition efficiency at 2.5 μM concentration for PC-3 and DU-145 cell lines, respectively. On the other hand, ζ-Stat demonstrated 52.6% and 57.6% inhibition efficiency for its 5 μM treatments. Results suggested that both compounds efficiently decline the *in-vitro* prostate cancer cell population at relatively mild concentrations providing the half-maximal inhibitory concentration (IC_50_) values are approximately 2.5 μM for ICA-1 T and 5 μM for ζ-Stat for both tested cell lines. Binding specificities against the respective targets for these inhibitors were confirmed based on cell-based assays and virtual screening and published in our previous reports [19,20,48,49]. Figure 6(c)-Figure 6(f) demonstrate the *in-vitro* cytotoxicities of two inhibitors based on WST-1 assay. Results indicated that both inhibitors provide mild toxicity to both prostate cancer cells lines which are significant (*P* < 0.05) for all tested concentrations with respect to their control samples. Our *in-vivo* results also suggested a solid association between aPKCs and Vimentin. In Figure 7, we demonstrate the *in-vivo* effects of aPKC inhibition using specific inhibitors on the expression of Vimentin in athymic nude mice xenografts.

Figure 7(a) demonstrates the representative images of excised subcutaneous tumors for PC-3 and DU-145 xenografts that were subcutaneously injected with ICA-1 T (80 mg/kg) and ζ-Stat (40 mg/kg). ICA-1 T treated PC-3 tumor volume was decreased by 56.7% (*P* < 0.05) while ICA-1 T treated DU-145 tumor volume was decreased by 62.6% (*P* < 0.05) with respect to their controls (Figure 7(a) and Figure 7(b)). ζ-Stat treatments did not show any significant effects as shown in Figure 7(a) indicating the inhibitor is not effective as ICA-1 T under tested *in-vivo* conditions. Figure 7(c) shows the immunohistochemical staining (IHC) for PKC-ι, PKC-ζ and Vimentin for PC-3 and DU-145 xenografts which were treated with ICA-1 T against its control. We only performed IHC for ICA-1 T treatments since it provided significantly better outcome as demonstrated in Figure 7(a) compared to PKC-ζ specific inhibitor ζ-Stat. ‘Histo’ score (H- score) measures the intensity of staining using percentage of stained cells. H-score for PKC-ι significantly (*P* < 0.05) reduced to 127 for the ICA-1 T treated samples for PC-3 xenografts compared to its control (H-score; 141). Similarly, H score was reported as 150 for DU-145 xenograft controls and the H score dropped significantly to 120 (*P* < 0.05) for ICA-1 T treated DU-145 xenografts. H score for PKC-ζ was not significantly changed for both controls and ICA-1 T treatments in both cell lines which strongly suggest that PKC-ζ was not affected by ICA-1 T treatments. Vimentin H score for control samples were 128 and 82 for PC-3 and DU-145 cell lines, respectively. Interestingly these scores dropped significantly (*P* < 0.05) to 102 and 34 for PC-3 and DU-145 in ICA-1 T treated tumors, respectively. These *in-vivo* results also indicate that Vimentin expression is largely affected as an outcome of PKC-ι inhibition. In addition, Figure 7(d) demonstrates the mRNA analysis of PKC-ι, PKC-ζ, Vimentin, E-cadherin, SNAIL1 and PRRX1 for the same subcutaneous tumors. As results indicated, mRNA levels of PKC-ι significantly decreased without changing PKC-ζ mRNA for the ICA-1 T treated samples. Similarly, Vimentin, SNAIL1 and PRRX1 mRNA levels significantly decreased for PKC-ι inhibited tumors with ICA-1 T. E-cadherin levels of DU-145 tumors increased significantly while PC-3 levels still reported higher than the control samples indicating ICA-1 T treatments following the same pathways that we explained in *in-vitro* analysis using RNA interference.

## Discussion

The role of atypical PKCs on different carcinomas has received contemporary attention [50]. aPKCs have two isoforms that are structurally and functionally distinctive: PKC-ι and PKC-ζ that are proactive in many cancers [51–54]. The proliferation of lung cancer cells depends heavily on the PKC-ι level by activating the ERK1 cascade [55]. PKC-ι overexpression plays a crucial role in the leukemia chemo resistance [56]. PKC-ι plays a key role cell cycle regulation of glioblastoma [57,58]. The development of non-small cell lung cancer is strongly dependent on PKC-ι [59]. aPKCs are known for the phosphorylation of Par6 thereby participates in TGF-β induced EMT [60]. Eder, *et al*. reported that increased expression of PKC-ι was associated with survival of ovarian cancers. In addition to our previous reports, PKC-ι overexpression is also noted because of its elite relationship with phenotype of transformed human melanoma *in-vivo* and *in-vitro* [19,20,35,61,62].

As shown in our previous reports, PKC-ι is massively responsible for melanoma progression and metastasis in particular [19,20,46,48]. Our *in-vitro* migration and invasion assays for human melanoma cells demonstrated that both invasion and migration were significantly reduced by PKC-ι inhibition by using the ICA-1 T specific inhibitor [20]. Moreover, we confirmed that PKC-ι stimulates Par6 and induced deprivation of RhoA to stimulate EMT in melanoma cells [19]. Additionally, as demonstrated by IP and IF and Western blot techniques, we revealed the solitary association of Vimentin and PKC-ι thereby phosphorylates Ser39 position of Vimentin [20].

On the other hand we have previously shown that both aPKCs are actively involved in prostate cancer cell survival [36]. But the metastatic process was not examined. In this article, we analyzed the connection of aPKCs in Vimentin dynamics thereby facilitating metastasis of PC-3 and DU-145 cells. Our results indicated that both aPKCs phosphorylate Vimentin at Ser33, Ser39 and Ser56 which makes aPKCs the most important component for Vimentin activation. Helfand and Mendez, *et al*. showed that phosphorylation at Ser39 increased the migration of fibroblasts [63,64]. Once phosphorylation takes place at Ser39, it leads to locally disassemble the VIF close to the edges and facilitate lamellipodium formation [63]. We analyzed this phenomenon in great depth by targeting multiple phosphorylation sites of the Vimentin structure which are crucial for increased motility. Initially we checked the outcomes of PKC-ι and PKC-ζ attenuation on prostate cancer cellular metastasis. As our results indicate (Figure 1(a)-Figure 1(d)), downregulated aPKC expression reduced the motility of DU-145 and PC-3 cell lines. We also showed that corresponding Vimentin levels in both cells were significantly decreased as a result of retardation of aPKC expression (Figure 1(e)). Interestingly, according to Westerns, expression of E-cadherin was upregulated in both DU-145 and PC-3 cell lines. Conversely, mRNA levels of E-cadherin did not change significantly but Vimentin mRNA levels decreased as a result of aPKC attenuation (Figure 1(g)). These results demonstrate the acquisition of more epithelial characters while reducing mesenchymal characteristics; which indicates slowing down of EMT process or acceleration of mesenchymal-epithelial transition (MET) in prostate cancer cells *in-vitro*. When EMT slows down, SNAIL1 and PRRX1 downregulation leads to stabilize the E-cadherin mRNA expression even though, the levels did not increase significantly and resulted a constant E-cadherin transcription. On the other hand, E-cadherin degradation reduces and thereby stabilizes the tight junctions between cells. This ultimately leads to an increase in E-cadherin protein levels available in the cell which is supported by stable mRNA production as a result of the repression of SNAIL1 and PRRX1. Upregulation of E-cadherin and downregulation of Vimentin expression helps cells gain apical-basal polarity which implies more epithelial characters. We conducted more Western blot experiments to analyze additional markers to further investigate EMT upon aPKC downregulation. Our results indicated that Smad2/3, Par6 and N-cadherin levels significantly decreased along with Vimentin while RhoA increased along with E-cadherin. It’s been illustrated before that aPKC/Par6 stimulates EMT when TGF-β receptors are activated. RhoA promotes actin stress-induced fiber formation and thus maintains epithelial cell integrity. TGF-β activation induces RhoA degradation leading to de-polymerization of F-actin (filament actin) and reduction of structural integrity resulting in a decrease in the adhesion between cells [60]. In addition, TGF-β induces SNAIL, a crucial transcription factor that stimulates EMT via the Smad cascades [65]. Epithelial cells lose apical-basal polarity during EMT, rearrange the extra cellular matrix (ECM), remodel the cytoskeleton, endure deviations in signaling programs that control cell shape maintenance, and alter gene expression to acquire an invasive mesenchymal phenotype that promotes individual cell motility [32]. Downregulation of epithelial genes such as E-cadherin to weaken intracellular tight junctions and upregulation of Vimentin and N-cadherin genes that assist mesenchymal phenotype is one of EMT’s key features. SNAIL1, SNAIL2, ZEB1, TWIST and PRRX1 are vital TFs in the EMT regulation. These TFs have highly specific expression profiles and are known to upregulate Vimentin expression during EMT in several carcinomas [39–44]. We have examined the effects of aPKC downregulation on said TFs but only SNAIL1 and PRRX1 expressions were altered due to *si*RNA knockdown of both aPKCs (Figure 2(a)). This indicates possible ties of SNAIL1and PRRX1 with aPKC activity in relation to EMT. To further analyze, we tested the effects *si*RNA knockdown of the expression of PRRX1 and SNAIL1 on the expression SNAIL1, PRRX1, both aPKCs and their degree of phosphorylation, E-cadherin and Vimentin (Figure 2(c)). Interestingly, downregulation of PRRX1 and SNAIL1 decreased the activated levels of aPKCs which are indicated by the degree of phosphorylation. These results indicate that SNAIL1 and PRRX1 has no responsibility on the expression of both aPKCs even though they showed a significant direct influence on the production of E-cadherin and Vimentin. It also confirms the strong relationship between aPKCs and Vimentin. It is evident that in a situation where less Vimentin is expressed (such as knocking down of SNAIL1/PRRX1), aPKCs activation is down regulated indicating less aPKCs are needed to drive the Vimentin dynamics. That was again the reason for decrease in SNAIL1 and PRRX1 levels as a result of aPKC attenuation as indicated in Western blots (Figure 2(a) and Figure 2(b)) and in qPCR experiments (Figure 2(e)). When less aPKCs are expressed, it weakens the transcriptional activity of SNAIL1 and PRRX1, which are crucial TFs which are responsible for upregulating Vimentin expression. The whole process initiated with attenuation of aPKCs, minimize the production of Vimentin which stabilize the E-cadherin as we observed in our results. The gene expression changes that contribute to epithelial phenotype repression and mesenchymal phenotype activation involve PRRX1 and SNAIL1. Their function is triggered in the initial phase in EMT, thereby playing a dominant role in cancer progression [39,44,66]. Since these transcription factors have unique expression profiles, their involvement in EMT rests on the type of cell or tissue involved and the signaling cascades that pledge EMT. During EMT, these cells downregulate E-cadherin expression [67]. In addition, cells begin to alter the genetic expression program to stimulate mesenchymal behavior. Repression of E-cadherin and upregulation of Vimentin is a hallmark of EMT. SNAIL1 and PRRX1 are two known TFs that upregulate Vimentin [39,44]. These TFs also regulate each other’s expression and interact functionally with target genes, and additional transcription factors also characterize the EMT transcription system guiding its progression. [39]. Our previous reports on melanoma confirmed that TGFβ stimulated Smad2/3 and Par6/PKC-ι/RhoA pathways stimulated the expression of PKC-ι along with Vimentin [19,20]. Regulation mechanisms which connects TGFβ with SNAIL1 and PRRX1 have been reported previously [32,44]. These results further confirm our experimental results of PRRX1 and SNAIL1 which ties with aPKC activation through Vimentin.

Preliminary experiments of co-IP (immunoprecipitation) of PKC- ζ and PKC-ι demonstrated a robust association with Vimentin in both DU-145 and PC-3 cell lines which was established with reverse-IP of Vimentin. Multiple microscopic methods were used to further investigate the association of aPKCs with Vimentin. Both IF and IG-TEM microscopic techniques revealed that Vimentin distributes along with both aPKCs both PC-3 and DU-145 cells. When aPKC expression was reduced as a result of *si*RNA treatments, Vimentin levels were also decreased. The degree of staining of Vimentin was decreased for the treated samples for both aPKCs, in addition to the targeted aPKC. We found that PKC-ι demonstrates a unique distribution pattern in PC-3 cell line compared to DU-145 cells. In IF experiments we noticed concentrated areas of PKC-ι close to the plasma membrane (Figure 3(a), top red panel) which was again observed in IG-TEM images (Figure 4(a), 6000X). Figure 4(b) shows an enlarged area of a PKC-ι concentrated area (tagged with 20 nm gold particles) which clearly shows that Vimentin (tagged with 10 nm particles) is also concentrated around PKC-ι. Both IF and IG-TEM techniques showed that these PKC-ι concentrated areas were markedly reduced for the PKC-ι *si*RNA treated samples (Figure 3(a), bottom red panel and Figure 4(c)). Even though PKC-ζ was not found to be concentrated along the plasma membrane, PKC-ζ distributed evenly throughout the cytoplasm of both cell lines along with Vimentin. These IF and IG-TEM results again confirm the association between Vimentin with aPKCs in these two prostate cell lines. Interestingly, the aggressive features such as formation of invadopodia, filopodia and lamellipodia were characteristically visible in control samples (Figure 3(a-Figure 3d), top panels), though they were not apparent in aPKC *si*RNA treated samples (Figure 3(a-Figure 3d), bottom panels). aPKC attenuation could harbored nuclei shrinkage and overall cell size reduction mainly through RhoA upregulation as observed in Figure 2(a) and Figure 2(b). These aPKC *si*RNA treated prostate cancer cells appeared to be unhealthy compared to respective control samples and also demonstrate a circular shape (epithelial characteristics) compared to elongated, spread cells in control samples (mesenchymal characteristics) (Figure 3(a-Figure 3d), bottom panels), which also supported the growth impedance we found upon aPKC attenuation in prostate cancer cells that resulted in a lower growth.

Once we established the association of aPKCs and Vimentin, we then tested whether Vimentin serves as a kinase target for both aPKCs. Ser6, Ser33, Ser39, Ser56, Ser71, Ser82 sites are very important phosphorylation sties in Vimentin structure, which often indicate the degree of Vimentin dynamics taking place in a cell. In addition to those said sites, other phosphorylation sites are known such as Ser4, Ser7, Ser41, Ser418, Ser429, Ser458, Ser457 but their exact involvement have not been well documented [18,23–25]. Ser6, Ser33, Ser39, Ser56, Ser71, Ser82 positions are situated in the Vimentin’s head region. We tested the role of aPKCs on several most important phosphorylation sites (Ser6, Ser33, Ser39, Ser56 and Ser71) that participate in Vimentin activation. As we shown in Western blots (Figure 5(a)), the degree of phosphorylation at Ser33, Ser39 and Ser56 positions were significantly reduced due to aPKC attenuation. But Ser6 and Ser71 sites did not show any effect as a result of aPKC depletion. Above data demonstrates that both PKC-ι and PKC-ζ are responsible for the phosphorylation of Vimentin in multiple phosphorylation sites and thereby controls the VIF dynamics in prostate carcinoma. All these aPKC phosphorylation targets locate on the N-terminal (head region) of the Vimentin structure (Figure 5(c)) which makes both aPKCs the two most important kinases for VIF dynamic process. Phosphorylation of the head region at these specific locations leads to the interconnection of individual chains in ‘unit length filaments’ (ULFs) to form long stable Vimentin filaments due to change of the charge in protein chains. VIF assembly and disassembly in prostate cancer cell lines is therefore a repeating process, mainly regulated by both aPKCs which are critical for prostate cancer cell motility as we confirmed through experimental results.

To further confirm the relationship of Vimentin with aPKCs we conducted *in-vivo* experiments using athymic nude mice xenografts. We used two novel aPKC specific inhibitors, ICA-1 T, a PKC-ι specific inhibitor and ζ-Stat, a PKC-ζ specific compound. Specificities against the respective targets for these inhibitors were confirmed based on cell-based assays and virtual screening and published in our previous reports [20]. Figure 6(a) and Figure 6(b) demonstrate the *in-vitro* dose curves for PC-3 and DU-145 cell lines, respectively. Data confirmed that both inhibitors are effectively decrease prostate cancer cell proliferation *in-vitro*. In addition, results also indicate that these two aPKC specific inhibitors provide mild-moderate toxicity to both PC-3 and DU-145 cells *in-vitro* regardless of the inhibitor concentration (Figure 6(c-Figure 6f)). Once proved the efficiency of these two compounds *in-vitro* then we went on to conduct in-vivo experiments on athymic nude mice. Figure 7(a) demonstrates the representative photographs of excised tumors for ICA-1 T and ζ-Stat for both tested cell lines. ICA-1 T treatments resulted a significant reduction of tumor volumes with respect to control samples but ζ-Stat did not demonstrate a significant reduction of the tumors (Figure 7(a) and Figure 7(b)). Figure 7(c) shows the immunohistochemical staining (IHC) for PKC-ι, PKC-ζ and Vimentin for PC-3 and DU-145 xenografts which were injected with ICA-1 T against its control. We only performed IHC for ICA-1 T treatments since it provided significantly better outcome as demonstrated in Figure 7(a) and Figure 7b compared to PKC-ζ specific inhibitor ζ-Stat. Having a significant reduction in H- score for PKC-ι in the ICA-1 T treated samples for PC-3 and DU-145 xenografts indicated that PKC-ι expression was markedly reduced in the tumors upon ICA-1 T treatments. Having an unaltered H score for PKC-ζ for ICA-1 T treatments indicate that PKC-ζ levels were not changed and it again confirms the PKC-ι specificity which was reported previously. The most interesting fact is that the H score of Vimentin in ICA-1 T treated samples significantly reduced by 20.3% (*P* < 0.05) and 58.5% (*P* < 0.05) for PC-3 and DU-145 cell lines, respectively with respect to controls. The reduction of PKC-ι in tumors were 9.92% and 20% for ICA-1 T treated tumors against control tumors for PC-3 and DU-145 xenografts, respectively. These *in-vivo* data confirms that ICA-1 T effectively inhibit PKC-ι thereby reduces its expression in tumors. Results also confirmed that PKC-ι associates with Vimentin proving the *in-vitro* data that we have discussed earlier in this report.

**Figure 8.**
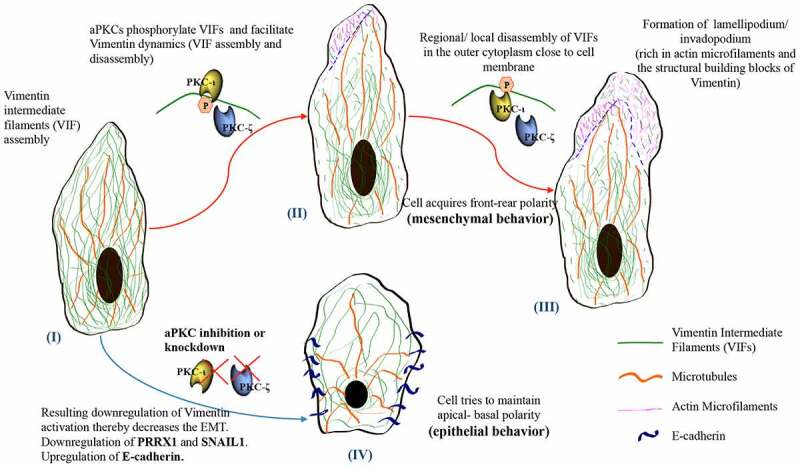
VIF assembly and disassembly depends on PKC-ι in prostate cancer cells. Events I, II and III show representative cartoons of three different stages of VIF dynamics for a prostate cancer cell that requires aPKCs for the optimal function. These representations illustrate mesenchymal features. Event IV shows a representative cell which lacked aPKCs (either treated with ICA-1 T/ζ-Stat or specific *si*RNA) to inhibit PKC-ι/ζ and the cell predominantly demonstrates epithelial features as a result of that aPKC diminution. VIF dynamics does not take place when aPKCs are not in optimal abundancy to govern the process smoothly thereby cells acquire more epithelial characteristics as evident in the results discussed in this manuscript

The animation in Figure 8 shows the VIF dynamics which take place in prostate cancer cells upon PKC-ι/ζ phosphorylation on Vimentin. As demonstrated in Figure 8-Ⅰ, 8-II and 8-III, VIF assembly and disassembly take place upon phosphorylation by PKC-ι/ζ. Figure 3 control samples provide experimental evidences of aPKC and Vimentin stains to support this argument and all the other experiments that we have conducted supported this claim. Vimentin stained panels in control samples of Figure 3 clearly shown the retraction of Vimentin network which indicate optimal VIF dynamics. Also aPKC stained panels (Figure 3- red panels in controls) indicated that abundant aPKCs are also available in these prostate cancer cells in which Vimentin displayed retraction of VIF network. Upon *si*RNA treatments, aPKC levels heavily decreased which resulted a downregulation of Vimentin and its dynamics, which was visible in *si*RNA treated samples in Figure 3 where no clear retraction of Vimentin was visible (green panels-treated). Also, as shown in Figure 3, both aPKCs heavily associate with Vimentin and upon *si*RNA knockdown of expression of PKC-ι and PKC-ζ, not only targeted aPKC expression but Vimentin expression in also decreased. In addition, we observed that the cells were constricted, rounded shape and tend to form cell colonies, which are emblems of epithelial phenotype behavior. As shown in the Figure 8 animation, microtubules are aligned to maintain the rear-front polarity by assembling along with Vimentin and once VIFs are phosphorylated, it retracts back allowing the cell matrix to undergo a propulsion of a pseudopodia and makes a lamellipodia or invadopodia. These lamellipodia are rich with actin microfilaments as shown in Figure 8-II and 8-III. In Vimentin rich conditions, they control the distribution of microtubules toward one direction to facilitate rear-front polarity. Involvement of microtubules and microfilaments are well explained in the introduction. However, once aPKCs are inhibited both aPKC and Vimentin expressions decrease and E-cadherin expression increases suggesting a slowdown or a possible reverse of the EMT process. When this phenomenon takes place, cells shape becomes rounded and cells tend to form colonies which are characteristics of epithelial phenotype. Our experimental results also demonstrated these changes such as rounded shape cells and colony formation (Figure 3, aPKC *si*RNA treated panels) rather than showing individual mesenchymal behavior as shown in control samples in Figure 3. Figure 8-IV shows a representative animation of aPKC inhibited prostate cancer cells with a relatively low Vimentin and higher E-cadherin expression and it also shows the apical-basal polarity which is essential for epithelial tissue formation. Our WB, IF, IHC and IG-TEM experiments supported and confirmed this argument.

For summary, many of the core aPKC regulatory functions of prostate carcinoma cells PC-3 and DU-145 could be regulated by association with and consequent phosphorylation of vimentin both *in-vitro* and *in-vivo*. In particular, Smad and Par6/aPKC/RhoA pathways promote EMT in PC-3 and DU-145 cells via SNAIL1 and PRRX1 which are crucial to keep optimal levels of activated aPKCs. Both aPKCs are upregulated simultaneously to facilitate EMT in tested prostate cancer cell lines to keep Vimentin dynamics in optimum conditions. This report also indicates that the use of particular inhibitors such as ICA-1 T and ζ-Stat not only decreases the survival of prostate cancer cells, but also adversely affects VIF dynamics thereby down-regulating EMT. Finally, this new model can be used to develop more specific and effective therapeutics for advanced prostate cancer patients based on PKC-ι/ζ and Vimentin. The findings here highlight significant therapeutic implications which merit further research.

## Supplementary Material

Supplemental Material
