# Supplementary figures and images for: Atypical PKCs activate Vimentin to facilitate prostate cancer cell motility and invasion
Source: Cell Adh Migr. 2021 Feb 11;15(1):37–57. doi: 10.1080/19336918.2021.1882782 (PMC7889213; doi:10.1080/19336918.2021.1882782)

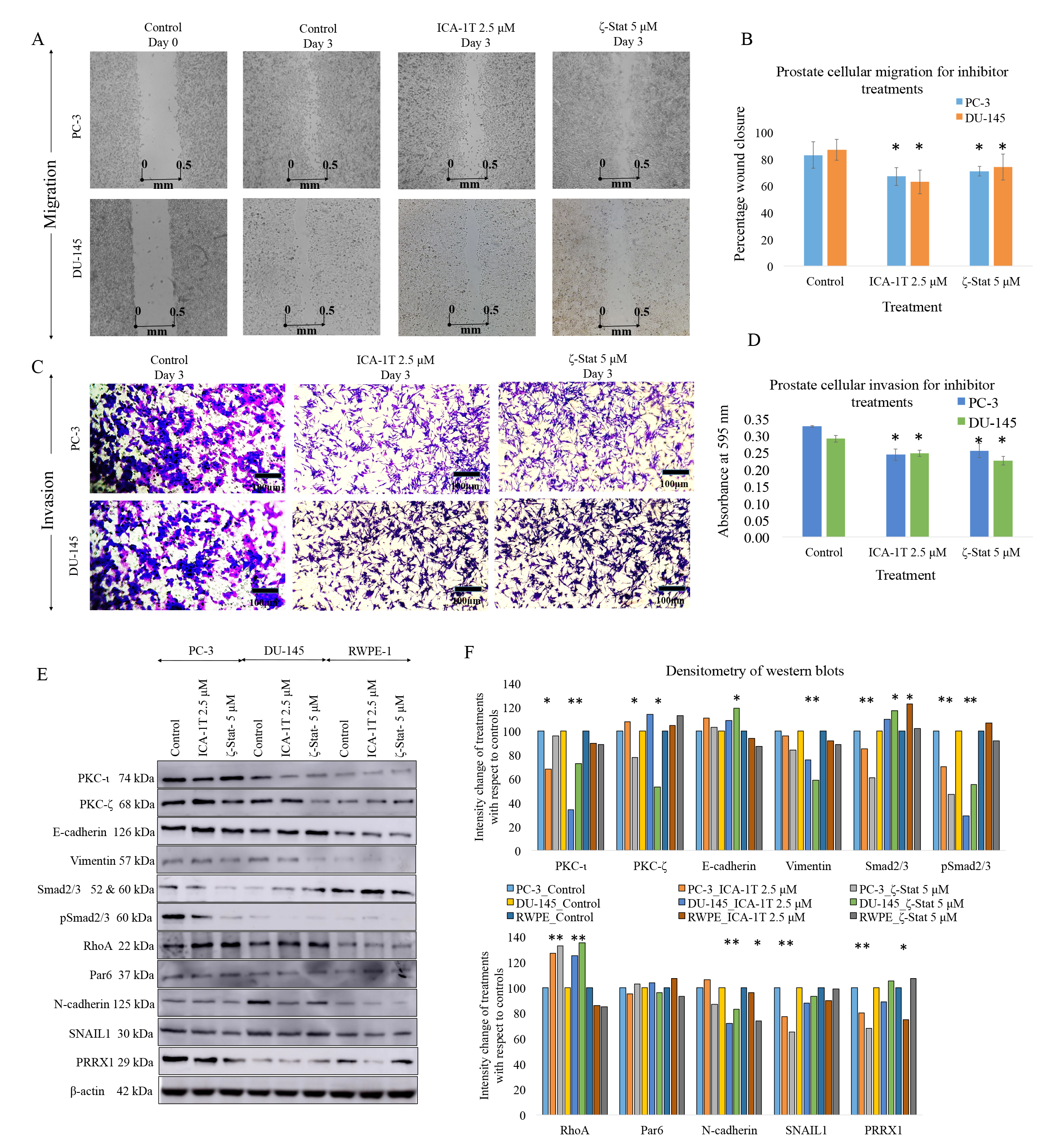

Supplement: Supplemental Material [file KCAM_A_1882782_SM5554.zip › Supplementary Figure 1.tif]

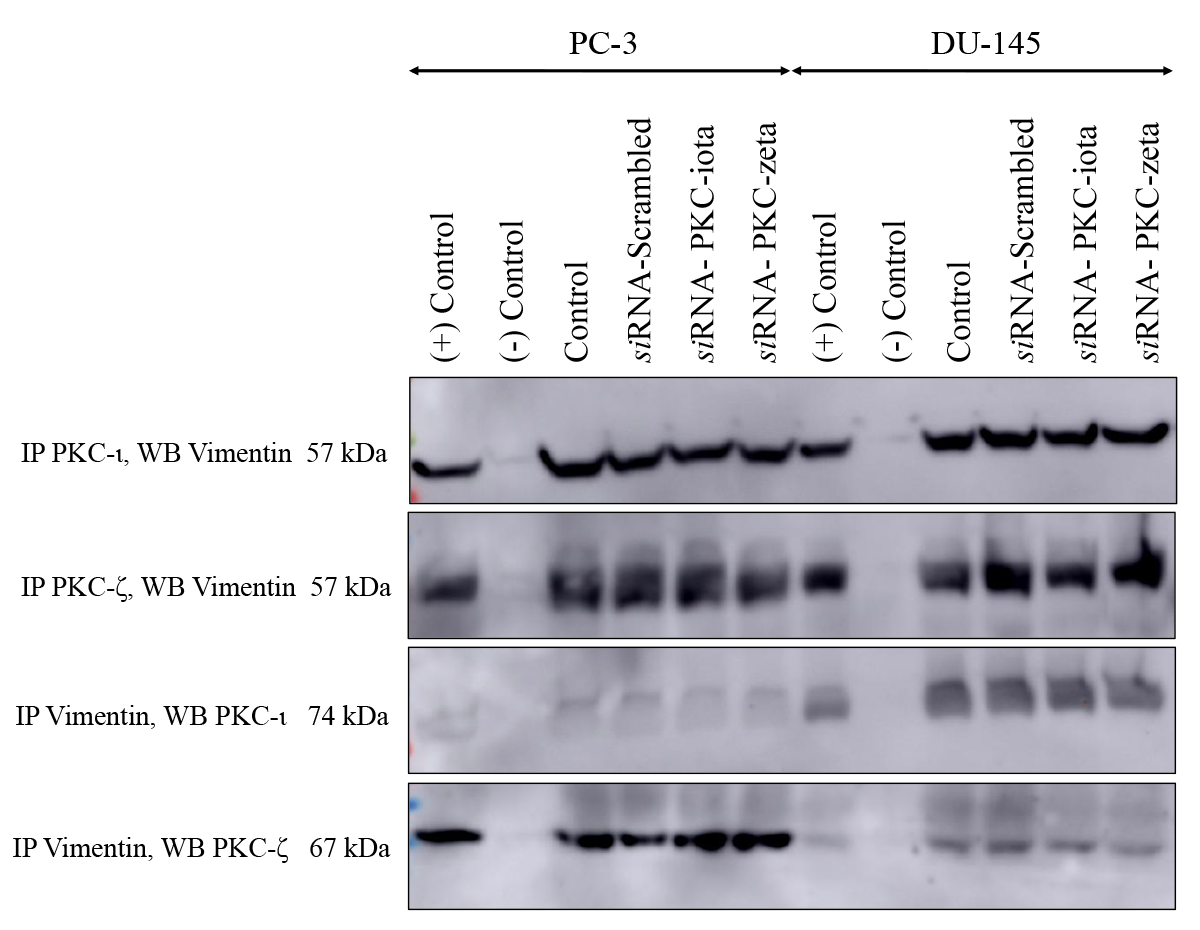

Supplement: Supplemental Material [file KCAM_A_1882782_SM5554.zip › Supplementary Figure 2.tif]
